# The first influenza pandemic in the new millennium: lessons learned hitherto for current control efforts and overall pandemic preparedness

**DOI:** 10.1186/1476-8518-7-2

**Published:** 2009-08-07

**Authors:** Carlos Franco-Paredes, Peter Carrasco, Jose Ignacio Santos Preciado

**Affiliations:** 1 Rollins School of Public Health, 550 Peachtree St. Mot 7th floor, Atlanta, GA 30308, USA; 2 Department of Immunization, Vaccines and Biological, World Health Organization Geneva, Switzerland; 3 Infectious Diseases and Clinical Immunology Unit, Department of Experimental Medicine, School of Medicine, Universidad Nacional Autónoma de México, Dr. Balmis 148, Col. Doctores, México

## Abstract

Influenza viruses pose a permanent threat to human populations due to their ability to constantly adapt to impact immunologically susceptible individuals in the forms of epidemic and pandemics through antigenic drifts and antigenic shifts, respectively. Pandemic influenza preparedness is a critical step in responding to future influenza outbreaks. In this regard, responding to the current pandemic and preparing for future ones requires critical planning for the early phases where there is no availability of pandemic vaccine with rapid deployment of medical supplies for personal protection, antivirals, antibiotics and social distancing measures. In addition, it has become clear that responding to the current pandemic or preparing for future ones, nation states need to develop or strengthen their laboratory capability for influenza diagnosis as well as begin preparing their vaccine/antiviral deployment plans. Vaccine deployment plans are the critical missing link in pandemic preparedness and response. Rapid containment efforts are not effective and instead mitigation efforts should lead pandemic control efforts. We suggest that development of vaccine/antiviral deployment plans is a key preparedness step that allows nations identify logistic gaps in their response capacity.

## Introduction

***"Miss M., Superintendent of Fordham Hospital, died yesterday of pneumonia following an attack of Spanish Influenza. The hospital is crowded with patients and short handed for nursing help. Miss M. had worked night and day until a week ago when she herself was stricken by the disease. Miss M. was 28 years old..." ***[1]

***"Mexico City, one of the world's largest cities, has closed schools, gymnasiums, swimming pools, restaurants, and movie theaters. Mexicans have donned masks for protection outdoors" ***[2]

Pandemics and epidemics of influenza viruses represent the most dramatic presentation of the rapid and effective spread of viruses among immunologically vulnerable human populations [3,4]. The rapidly evolving nature of influenza viruses has profoundly impacted humankind [5]. Fear and anxiety associated with influenza epidemics flourish on uncertainty due to their often unpredictable course and ultimate outcome. As a result of the dynamic and relentless evolutionary struggle between humans and influenza viruses, effective public health interventions demand an active adaptation and strengthening of responses and preparedness plans [6,7].

At this moment in time, the World Health Organization (WHO) has raised this outbreak to a category of a moderately severe influenza pandemic [6]. Since the 1968 Hong Kong pandemic, this is the first declaration of an influenza pandemic in 41 years. This pandemic highlights the perennial threat of Influenza viruses. Thus, it is critical to apply the lessons learned from previous pandemics and those learned up to now, from the ongoing influenza A(H1N1)v pandemic in 2009.

### Lessons learned for strengthening influenza preparedness and response

#### 1) Overall preparedness plans

**Figure 1:**
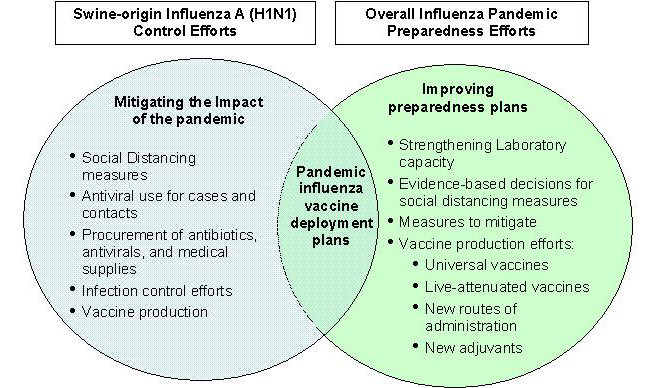
**Applying lessons learned from the ongoing influenza A (H1N1) pandemic to control efforts and overall influenza pandemic preparedness**.

The first and foremost important lesson from the current pandemic is that we need to focus our planning and response efforts on those interventions that are critical during the early phases of a pandemic, when there is no availability of pandemic vaccine [5]. Responding to the current pandemic or preparing for future ones, nation states need to develop or strengthen their laboratory capacity for influenza diagnosis; and should begin augmenting their stockpiles of antivirals and antibiotics, as well as begin preparing their vaccine/antiviral deployment plans (Figure 1).

All governments need to prepare and/or respond to the current influenza A(H1N1)v pandemic. It is therefore crucial to evaluate current response capacities: a) hospital surge; b) pharmaceuticals; c) social distancing measures/communications protocols; d) case management and surveillance activities; e) deployment plans to move people, medical supplies, and pharmaceuticals (vaccine, antivirals, antibiotics, etc) and available syringes; f) revise guidelines for priorization of vaccine use.

#### 2) Improving laboratory diagnostic capacity for influenza diagnosis

Given that Mexico became the epicenter of the current influenza epidemic, it is important to note that Mexican authorities acted in a timely, transparent, and effective manner to control the outbreak and notify international public health authorities despite its limitations in laboratory capacity. In this regard, international collaboration by Mexican, Canadian, and American scientists led to the rapid identification of the influenza A(H1N1)v strain leading to the early institution of aggressive social distancing interventions. However, this outbreak demonstrates that need for improved laboratory capacity and the strengthening or expansion of laboratory networks for influenza testing to include resource-limited settings. This is a critical policy step to achieve the early confirmation of an outbreak with potential pandemic spread [8-10]. The collaborative international laboratory networks that facilitated the identification of the current pandemic strain are not currently in place in many regions of the world where an influenza pandemic may erupt.

#### 3) Considering the epidemiology of previous pandemics

By June 11, 2009, 74 nation states have cases, with approximately 27,737 confirmed cases and 141 deaths leading WHO to raise the outbreak to a phase 6 [4]. The influenza A(H1N1)v strain has been associated with an overall low transmissibility and low case-fatality rate in Mexico (0.6%) [5]. The estimated transmissibility of the infection (R_0_) ranges from 1.4 to 1.6 which is higher that of seasonal influenza and lower than the three previous pandemics [9]. Epidemiologic patterns in the novel influenza A(H1N1)v outbreak have consistently shown the disease taking its hardest toll on younger people [9-13]. In the United States, 64% of the novel flu cases have occurred in the 5- to 24-year-old age-group [14]; and in Mexico in the group of 15 to 50. A potential explanation for this epidemiologic distribution maybe that adults, especially those older than 60, appear to have some cross-antibody response to the pandemic strain [14].

While we cannot predict the events during the upcoming 2009 winter months of this pandemic, so far this pandemic is relatively mild in comparison to the 1918, 1957, and the 1968 pandemics. However, in facing the current influenza A(H1N1)v pandemic, there are epidemiologic similarities between the 1918–1919 influenza pandemic and the onset of the 2009 influenza A(H1N1)v that and unavoidable and need to be considered. That said, it is also important to recognize the significant social, cultural, political, and scientific differences that do exist between that period and the current worldwide order (Table 1) [5].

**Table 1:** Comparison of the 1918–1919 and the 2009 H1N1 influenza pandemics

	**1918–1919**	**2009**
**Influenza virus**	Avian Influenza A H1N1	Swine-Origin-Influenza A(H1N1)v

**Social and political Context**	World War I – U.S. troops being deployed to Europe	One of the largest economic recessions in the U.S. with worldwide reach
		Globalization, ease of travel, population overgrowth, megacities

**Source of viral strain emergence**	Historians have suggested to potential origins for this pandemic viral strain in China or in the Midwestern US military camps during World War I	Unclear source, phylogeny of the virus demonstrates to be an Eurasian H1N1 swine strain

**Seasonality and transmissibility**	Highly-transmissible – three succeeding waves of the outbreak	Cases surfaced in early spring in Mexico City and in California, U.S.A.
	Initial wave spring 1918 with sustained multifocal transmission	Sustained transmission (two generations) only in North America

**Affected age groups**	Most deaths occurred within the first six months of the pandemic.	Most deaths occurred within a three week time span.
	Most affected group 15–34 year-old population	Most affected group is the 5 to 30; case-fatality rate has ranged from 5 to 45 years of age

**Case management**	Insufficiency of healthcare systems	Wider availability of healthcare institutions
	Absence of effective antimicrobials for treating secondary bacterial pneumonias.	Availability of broad-spectrum antimicrobials for treating secondary bacterial pneumonias
	Medical intensive care in early phases of development	Sophisticated medical intensive care and mechanical ventilatory support
	Insufficient infection control activities	More established infection control activities and programs

**Virulence**	Highly virulent	Virulence only demonstrated as causing most fatalities in Mexico

**Availability of vaccine**	No	No

**Susceptibility to antivirals**	No availability of antivirals	Susceptibility to neuraminidase inhibitors (oseltamivir). However, there are growing number of resistant viral strains to oseltamivir

**Nosocomial transmission**	Highly transmissible in hospital settings	Possibility of nosocomial transmission under investigation with 81 healthcare workers affected in the U.S [23]

**Molecular characterization**	H1N1 avian strain without evidence of reassortment (4)	H1N1 (triple reassortant – human – avian – swine)

**Natural history of the outbreak and outcomes**	More than 300 million cases worldwide	By June 11, 2009, 74 nation states have cases, with approximately 27,737 confirmed cases and 141 death
	More than 50 million people deaths worldwide	

The major concern of this pandemic remains the case-fatality rate seen among young Mexicans, which continues to be largely unexplained but may potentially be attributed to an exuberant inflammatory response or interferon antagonism among young individuals compared to those in extremes of life, as has been suggested to have occurred during the 1918–1919 pandemic [6]. This phenomenon needs to be elucidated, and in this regard there are ongoing efforts aimed in deciphering the underlying pathogenesis associated with these deaths. In addition, preliminary clinical observations have suggested that those young Mexicans who happened to be receiving lipid-lowering drugs of the statins class (for other indications) during influenza A(H1N1)v infection had better outcomes relative to those not receiving these drugs [Jose Santos-Preciado, personal communication]. While we do not have solid data to illustrate this anecdotal experience, we believe that the potential use of anti-inflammatory drugs in the setting of a pandemic to ameliorate the clinical severity and improve clinical outcomes in countries without enough supply of antiviral drugs or available pandemic vaccine merits further research [15].

#### 4) Rapid containment strategies vs. mitigation strategies

Nowadays, with both easy access to global travel and high population density, rapid containment of influenza epidemics is almost impossible to conceive [16]. Moreover, the current 2009 influenza A(H1N1)v pandemic definitely illustrates that we cannot over-rely on the rapid availability of a pandemic influenza vaccine (16). Most control efforts should therefore ensure that preparedness and response plans are in place to mitigate high levels of morbidity and mortality; and the social and economic disruption that can be expected during the early phases of a pandemic.

In this sense, the WHO strategic plan for influenza is intended to ensure that measures are in place to mitigate the high levels of morbidity and mortality as well as the social and economic disruption that can be expected during the next pandemic [17,18]. A few of the relevant strategic actions contemplated by WHO have included: strengthening the early warning system; to intensify the rapid containment operations, building additional capacity to cope with a pandemic, and coordinating global scientific research and development activities [17]. It has been proposed that within rapid containment strategies, the main goal is to stop the development of pandemic influenza when it is initially detected and before the virus has been able to spread widely [19].

Despite the plans of WHO and national governments, the current influenza A(H1N1)v demonstrates that rapid containment strategies are largely ineffective and logistically unfeasible. The main reasons behind the lack of efficacy of rapid containment operations are multiple. There is limited laboratory capacity in most settings of the world, where influenza pandemics may suddenly originate such as the case of Mexico. Additionally, there is an overall lack of logistic planning and support to rapidly deploy vaccine, antivirals, and even medical supplies.

In a similar manner, only a minority of nation states have wide availability of reserve stockpiles of antivirals, antibiotics, and personal protective equipment for infection control purposes. Even deployment of antivirals from the WHO antiviral stockpile may not arrive in affected areas immediately after the identification of a pandemic in a timely fashion to prevent its rapid spread. Antiviral drugs, particularly the neuraminidase inhibitors, are most effective when given early, and must be administered early in the clinical course if they are to truly have an impact in shortening the time course of the disease and likely the spread of the virus from person to person [17]. To illustrate this point, the 1918–1919 influenza pandemic sickened more than 800 million worldwide [3,8]. Despite the much larger current global population, a rough tally of all stockpiles of antiviral drugs indicates there are only 250 million courses of antivirals currently available [20]. Moreover, given the insufficient stocks of antivirals in most settings of the world, ineffective or delayed use of antivirals in attempting rapid containment strategies may lead to rapid development of antiviral resistance [20]. Currently, WHO is redrafting their guidelines with regard to the most effective use of antiviral drugs. These guidelines will focus on avoiding their indiscriminate use and therefore preventing speeding the spread of neuraminidase inhibitor resistance which has been currently identified in many settings. In addition, these new guidelines will likely distinguish prophylaxis vs. treatment, with an emphasis on prophylaxis, and avoiding the use of antiviral drugs for the purpose of curbing the viral spread of influenza viruses.

History teaches the great value of non-pharmacological interventions against influenza pandemics, and these measures have immediate applicability [13,15]. Such interventions include the timely application of social distancing measures during the initial stages of an epidemic when there is limited information on the biology of the pandemic virus (Figure 1). More evidence-based data are required to optimize decision-making ability of policymakers in terms of the efficacy of social distancing measures. However, early evidence from the Mexican outbreak indicates that prohibiting mass gatherings was instrumental in preventing further spread of the outbreak [5].

#### 5) Pandemic Influenza vaccine production

Current world capacity to produce influenza vaccines is around 700 million to 900 million doses annually, which would translate into between at least one billion to two billion doses of monovalent influenza A(H1N1)v pandemic vaccine if the decision is made to switch from production of seasonal influenza vaccine [21]. At this point in time, the key question centers on the possibility of developing and producing a monovalent vaccine [5,22].

In this regard, it has been estimated that in 2009, seasonal influenza vaccine production worldwide is approximately 480 million doses [22]. This number is in response to the relatively historically low demand for manufacturing of the vaccine. In 2006, WHO urged nation states to introduce seasonal influenza vaccine into their national routine immunization plans as a public health priority or to increase their use for those nation states with existing use of seasonal vaccines [21]. Since then, a growing number of nation states have stimulated vaccine demand most likely due to the increasing recognition of the significant burden of diseases caused by seasonal influenza in the Northern and Southern hemispheres. Another important issue to consider in the biology of the current influenza A(H1N1)v strain is the potentially evolving (drifting or shifting) nature of this agent, and therefore it is unclear at this point whether the current vaccines will be effective and safe.

Short term prospects for producing a larger number of pandemic influenza vaccine doses remains limited. Fear of a pandemic has rushed health officials and politicians to protect their own citizens without working in cooperation with other nation states in procuring pandemic influenza vaccine. The rest of the world awaits a decision by the WHO and major pharmaceutical companies [22]. Strong international collaborative efforts are critical in this era of globalization with regards to influenza vaccine production efforts. Experts are advocating new vaccine adjuvants, an intradermal route of administration to optimize amounts of vaccine, and new vaccine production strategies such as mammalian or insect cell culture to accommodate a larger number of influenza vaccine, as well as the search for universal vaccines that would potentially offer the best cross-protection against different influenza strains [21,22]. A final consideration in regards to pandemic influenza vaccine policy-making is to consider the experience of the Panamerican Health Organization revolving fund for procuring and purchasing vaccines. This type of international collaboration would be the next step to have available pandemic influenza vaccines for most areas of the world.

In the meantime, nation states should begin planning for pandemic influenza vaccine deployment or antiviral deployment regardless of the current absence of availability of pandemic influenza vaccine [23-25]. The WHO guidelines build on the premise that each Member State has drawn up an overall influenza pandemic preparedness plan that includes a deployment plan for the activities involved in delivering a pandemic influenza vaccine (within seven days of the time it is made available to the country). This seven-day time frame should be respected in order to protect individuals as quickly as possible, to reduce disease transmission and to take advantage of the power of vaccine to fight the disease. The successful eradication of smallpox and efforts to eradicate poliomyelitis in many regions of the world have operated on this principle (Figure 1). Furthermore, developing a deployment plan allows the identification of human resources, medical supplies, and logistic gaps prior to the occurrence of a large number of cases or deaths prior to a pandemic.

In summary, we should continue to learn and effectively apply the lessons we learn from this unfortunate influenza A(H1N1)v pandemic. Theultimate goal of continuously improving pandemic influenza preparedness is to identify those policy and planning structures and processes that could withstand the test of time to prepare and respond to *any *potential health-focused emergency or natural disaster.
